# Dopamine receptor 1-expressing cells in the ventral hippocampus encode cocaine-context associations

**DOI:** 10.21203/rs.3.rs-8079719/v1

**Published:** 2025-11-25

**Authors:** Veronika Kondev, Arthur Godino, Brian T. Kipp, Evelyn Hennigan, Clara Casey, Vishwendra Patel, Sarah Naguib, Adam Ripp, Jacob Abroon, Liz Kahn, Isla Racine, Earnest P. Chen, Clementine Blaschke, Angelica Minier-Toribio, Molly Estill, Alexa LaBanca, Li Gan, John F. Fullard, Panos Roussos, Eric J. Nestler

**Affiliations:** 1 Nash Family Department of Neuroscience & Friedman Brain Institute, Icahn School of Medicine at Mount Sinai, New York, NY 10029, USA; 2 Aix-Marseille Université, INSERM, INMED, Marseille, France; 3 Department of Psychiatry & Friedman Brain Institute, Icahn School of Medicine at Mount Sinai, New York, NY 10029, USA; 4 Department of Genetics and Genomic Sciences & Icahn Genomics Institute, Icahn School of Medicine at Mount Sinai, New York, NY 10029, USA; 5 Center for Disease Neurogenomics, Icahn School of Medicine at Mount Sinai, New York, NY 10029, USA; 6 Helen and Robert Appel Institute for Alzheimer’s Disease Research, Brain and Mind Research Institute, Weill Cornell, New York, NY USA; 7 Mental Illness Research, Education and Clinical Centers, James J. Peters VA Medical Center, Bronx, NY 10468, USA; 8 Center for Precision Medicine and Translational Therapeutics, James J. Peters VA Medical Center, Bronx, NY, USA.

## Abstract

Dopamine (DA) signaling facilitates emotional valence, reward prediction error, and motivation; thus, dysfunctional dopamine signaling has been tied to several neuropsychiatric disorders, including substance use disorder (SUD). While most research has focused on striatal DA signaling, we and others have established that the ventral hippocampus (vHPC) also exhibits topographical organization of mostly non-overlapping dopaminoceptive neuronal populations: those expressing the DA receptor type 1 (D1) or type 2 (D2). Here, we use fiber photometry, optogenetics, and single nuclei RNA-sequencing (snRNA-seq) to explore how dopaminoceptive cells in the vHPC control cocaine-context memory expression. We reveal that vHPC D1 cells are inhibited by cocaine-conditioned contextual cues and that this inhibition is necessary and sufficient for reward-context associations driving cocaine conditioned place preference (CPP). Meanwhile vHPC D2 cells are not dynamically altered by learning but rather support positive reinforcement. Transcriptional changes induced by cocaine CPP encode these distinct functions, with D1 cells undergoing the greatest gene expression changes tied to synaptic signaling and plasticity. Together, these studies broaden our understanding of the role of DA within the larger mesocorticolimbic system and reveal new neuronal and circuit mechanisms underlying how drugs of abuse promote strong associative learning that could drive compulsive drug seeking characteristic of SUD.

## Introduction

Associative learning enables organisms to adapt their behavior based on relationships between environmental cues, emotional states, physiological needs, and motivational outcomes^1,2^. Dopamine (DA) plays a critical role in this process by encoding salient information including reward, aversion, or prediction errors, and then transmitting it to downstream targets to shape learning and behavior^3–5^. While most studies have centered on DA’s actions in the striatum, particularly through its activation of distinct DA receptor (type 1 [D1]- and type 2 [D2])-expressing medium spiny striatal neurons^6–12^, less is known about how DA influences associative learning processes in other limbic regions.

The ventral hippocampus (vHPC), a brain region essential for emotional regulation and context encoding, is increasingly recognized as a key player in motivated behavior and affective learning^13^. Notably, the vHPC– like the striatum – contains distinct, mostly non-overlapping populations of D1 or D2 neurons, which differ in their anatomical and transcriptional identity and play opposite roles in regulating exploratory behavior^14–16^.

Here, we define the functional contributions of these two distinct vHPC dopaminoceptive neuronal populations to associative learning in the context of cocaine reward. We reveal that vHPC D1 cell inhibition is necessary and sufficient to promote cocaine conditioned place preference (CPP), suggesting that DA activation of D1 cells constrains associative learning. In contrast, vHPC D2 cells are preferentially activated by unconditioned stimuli and support positive reinforcement, indicating that DA inhibition of these cells too opposes outcome-driven motivation. Together, these findings identify dissociable roles for vHPC D1 and D2 neurons in processing context-reward relationships and valence-specific motivational signals. This work expands our understanding of how limbic DA-sensing circuits regulate associative learning beyond the striatum.

### Noncontingent acute cocaine exposure increases FOS levels in D1 vHPC cells

To first elucidate how drugs of abuse affect vHPC D1 and D2 neuronal activation, we assessed FOS levels following an acute injection of cocaine (20 mg/kg) or saline in male and female mice. We visualized D1- and D2-expressing cell bodies in the vHPC by crossing D1-Cre and D2-Cre lines to eGFP::L10a^fl/fl^ reporter mice, which we have previously validated recapitulate endogenous *Drd1* or *Drd2* transcripts in the vHPC^16^. Using *Cellpose* deep learning framework for cellular segmentation of D1- or D2-GFP+ and FOS+ expression, we validated topographical organization of D1 versus D2 cells within distinct layers of the vHPC consistent with our previous report (Fig. S1A)^16^; for example, D1 cells were enriched in the ventral CA3 (vCA3) pyramidal layer and ventral dentate gyrus (vDG) granule cell layer (gc), while a higher density of D2 cells was observed in the ventral subiculum (vSub), ventral CA1 stratum lacunosum-moleculare (vCA1 slm), and vDG polymorph layer (Fig. S1).

**Figure 1.**
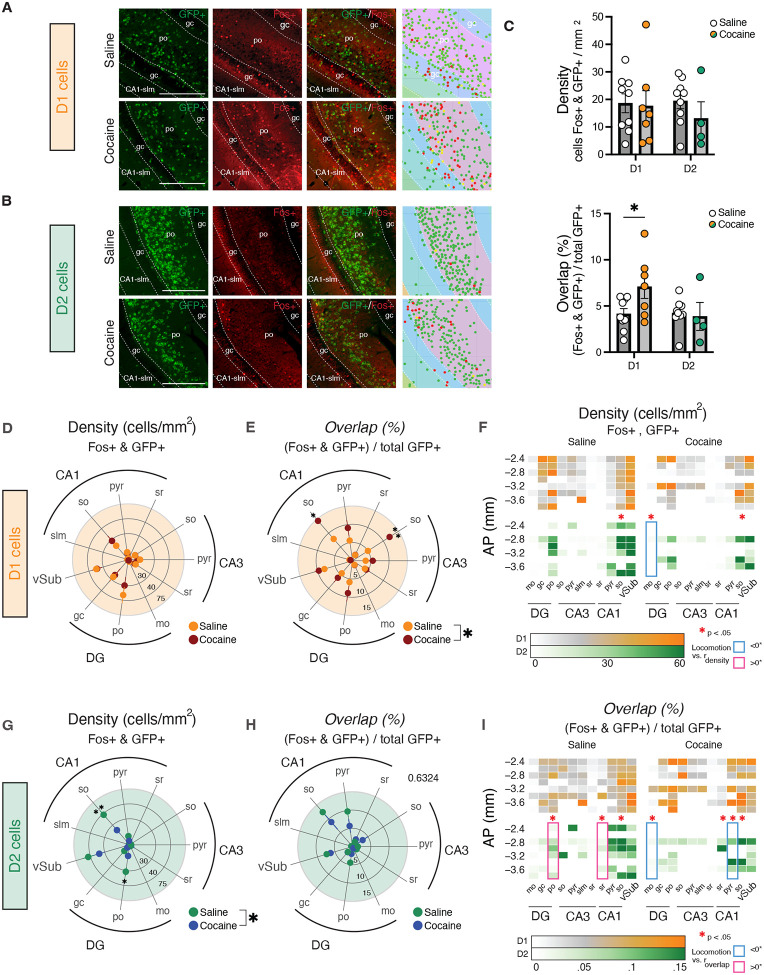
Acute cocaine exposure increases FOS expression in vHPC D1 cells. A) Representative photos of FOS expression in D1-GFP+ cells. Scale bar represents 350μm. B) Representative photos of FOS expression in D2-GFP+ cells. Scale bar represents 350μm. C) Density of co-expressing FOS and GFP in D1 and D2 mice (top); proportion of FOS+ and GFP+ cells (bottom; P=0.0206, 2-Way ANOVA). D) Density of FOS+ & GFP+ D1 cells across subregions in saline- versus cocaine-treated mice (Sal, *n=*9; Coc, *n*=7). E) Proportion of co-labeled FOS+ and GFP+ cells out of total D1-GFP+ cells (P_Drug_=0.02227, 2-Way ANOVA). F) Density of GFP+ and FOS+ cells along AP axis of vHPC and correlations with locomotor activity. G) Density of FOS+ and D2-GFP+ co-expressing cells across layers (P_Drug x Region_ = 0.01856, 2-Way ANOVA) (Sal, *n*=9; Coc, *n*=4). H) Proportion of co-labeled D2-GFP+ and FOS + cells out of total D2-GFP cells. I) Proportion of FOS and D1 or D2 GFP co-expression along AP axis with correlations to locomotor activity. Data represented as mean ± s.e.m

We assessed co-localization of D1-GFP or D2-GFP with FOS and observed that acute cocaine exposure significantly increased FOS+ expression in D1, but not D2, cells, indicating cell-type-specific activation (Fig. 1A–C). Virtually all FOS expression was neuronal, with no effect of cocaine exposure on general FOS activation in non-DA receptor expressing cells (Fig. S1D). Upon segmentation of the vHPC into distinct layers, we observed that this increase in co-localization between D1-GFP and FOS was most heavily found in stratum oriens (so) layers of the vCA1 and vCA3. While we found no general changes in D2-GFP FOS co-expression, we did observe that cocaine exposure significantly downregulated FOS in D2-GFP+ cells selectively in the vCA1-so layer, suggesting opposite effects on D1 versus D2 cells in this specific area. These opposite effects of cocaine *in vivo* are consistent with prior electrophysiological findings with D1 versus D2 receptor agonists on D1 and D2 cell activity in vHPC slices *ex vivo*^15^. Finally, we observed that D2-GFP–FOS co-expression significantly correlated with cocaine-induced locomotion. Together, these data indicate cell-type-specific activation of dopaminoceptive vHPC cells by acute cocaine. Given that the most robust effects were observed in so layers, these data also point at interneurons as a key primary target of acute cocaine action in the vHPC.

### Cocaine-context learning inhibits vHPC D1 cell activity

**Figure 2.**
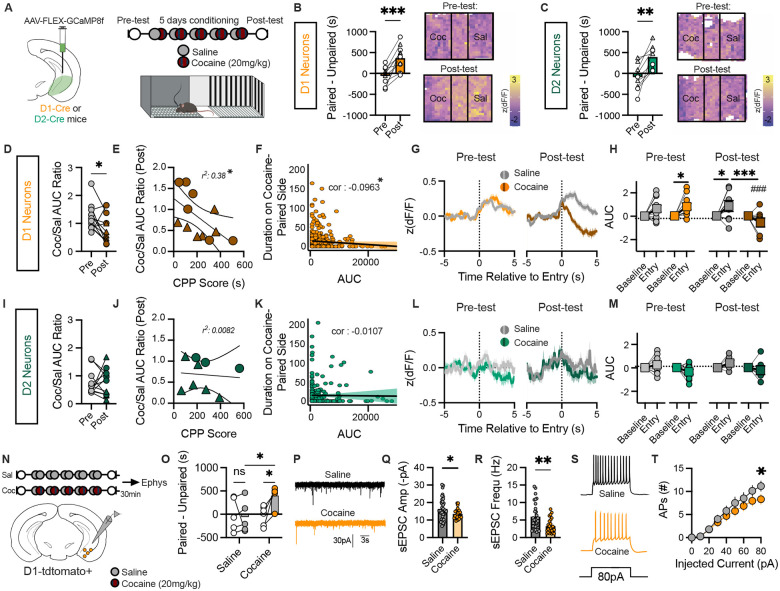
vHPC D1 cell activity is inhibited following cocaine-context learning. A) Viral schematic and cocaine CPP paradigm. B) Preference in D1-Cre mice (*n*=12) and subsequent representative heat map of ΔF/F in D1 cells in CPP apparatus before and after conditioning (P=0.0003, Paired t-test); triangles represent females and circles represent males. C) Preference in D2-Cre mice (*n*=9) and representative heat map of ΔF/F in D2 cells (P=0.0023, Paired t-test); triangles represent females and circles represent males. D) Change in D1 activity discrimination on cocaine versus saline paired side across conditioning; triangles represent females, circles represent males (P=0.0433, Paired t-test). E) Correlation of D1 discrimination and CPP score (P=0.0248, Linear regression). F) Correlation of D1 activity and time spent on cocaine-paired side during post-test. G) Change in D1 cell activity upon entry to saline or cocaine paired side. H) Area under the curve (AUC) upon entry (P_Entry x Post-test x Side_ = 0.01115, 4-Way ANOVA). I) D2 discrimination scores; triangles represent females, circles represent males. J) Correlation of D2 discrimination and CPP score. K) Correlation of D2 activity and time spent on cocaine-paired side during post-test. L) Change in D2 cell activity upon entry to saline or cocaine-paired side. M) Resulting AUC upon entry. N) Schematic of electrophysiology recordings following saline (control, *n*=5) or cocaine conditioned mice. O) Resulting preference during pre-test and post-test (P_Cocaine x Post-test_ = 0.0470, 2-Way ANOVA). P) Representative spontaneous excitatory postsynaptic currents (sEPSCs) from saline versus cocaine CPP mice. Q) sEPSC amplitude (P=0.0448, Unpaired t-test). R) sEPSC frequency (P=0.0027, Unpaired t-test). S) Representative action potentials (APs) elicited with 80 pA current injection. T) Number of APs elicited across increasing current injections (P=0.0424, 2-Way ANOVA). Data represented as mean ± s.e.m

Given the role of the vHPC in modulating cocaine learning^17–21^, we next determined how cocaine-context associations alter vHPC D1 and D2 neuronal activity *in vivo*. We performed fiber photometry recordings from D1 or D2 vHPC cells *in vivo* across cocaine conditioned place preference (CPP) in male and female mice (Fig. 2A). After conditioning, D1, but not D2, neurons exhibited a significant reorganization in neural activity between cocaine- and saline-paired compartments (Fig. 2B–C; S2A, S2D). This resulted in enhanced discrimination between contexts in D1 neurons, evident as a decrease in cocaine-context versus saline-context elicited D1 activity (Coc/Sal AUC Ratio) (Fig. 2D). Differences in context-induced D1 activity significantly correlated with CPP score, with less D1 cell activity on the cocaine-side (higher discrimination) associated with greater CPP expression (Fig. 2E–F). Entry into the cocaine-paired context also evoked inhibition of D1 neurons, that emerged only during CPP expression (post-test) (Fig. 2G–H). We did not observe any significant changes in activity upon exiting either compartment (Fig. S2B-C). In contrast, D2 neurons showed no learning-dependent reorganization or cocaine- or context-evoked responses (Fig. 2I–M; S2D-F).

To probe possible synaptic mechanisms underlying the decrease in activity of vHPC D1 neurons we observe during cocaine-context memory recall, we conducted *ex vivo* whole-cell patch clamp recordings from vHPC D1 neurons in brain slices 30 min after CPP testing in cocaine- and saline-conditioned mice (Fig. 2N–O). Cocaine conditioning significantly reduced excitatory synaptic input of both spontaneous excitatory postsynaptic current (sEPSC) amplitude and frequency, resulting in decreased cell excitability (Fig. 2P–T; Fig. S3A-B). This was not associated with any changes in passive membrane properties, including resting membrane potential or threshold potential (Fig. S3C-D). Importantly, synaptic effects were absent in mice that did not form a cocaine preference (Fig. S3E-H). Together, these findings reveal that cocaine-context memory expression selectively decreases vHPC D1 activity via reduced excitatory transmission, identifying potential circuit mechanisms for context-dependent drug seeking.

### vHPC D1 cells undergo learning-dependent plasticity following aversive conditioning

We next confirmed that vHPC D1 cells are modulated by associative learning across contexts using fiber photometry in conjunction with fear conditioning and recall (Fig. S4A). Mice were exposed to 6 tone (CS+)-shock (US) pairings during conditioning, and then 24 hours later placed back into the same context and exposed to the tone (6 CS+), this time in the absence of shock. Consistent with our prior report, both D1 and D2 vHPC cells responded to footshock during conditioning (Fig. S4B-D)^16^. During recall, only D1 neurons exhibited responses to the shock-paired tone (Fig. S4E-F).

These findings indicate that D1 neurons are activated by aversive cues (shock-paired tone), but inhibited by rewarding contextual cues, suggesting that vHPC D1 activity may encode the valence of a stimulus. Conversely, vHPC D2 cells showed no learning-dependent plasticity across fear conditioning and recall (Fig. S4F). These results highlight selective and bidirectional modulation of D1 neurons by emotionally salient stimuli, supporting a role for D1, but not D2, cells in encoding learned valence across behavioral paradigms.

### vHPC D1 cells control cocaine CPP

**Figure 3.**
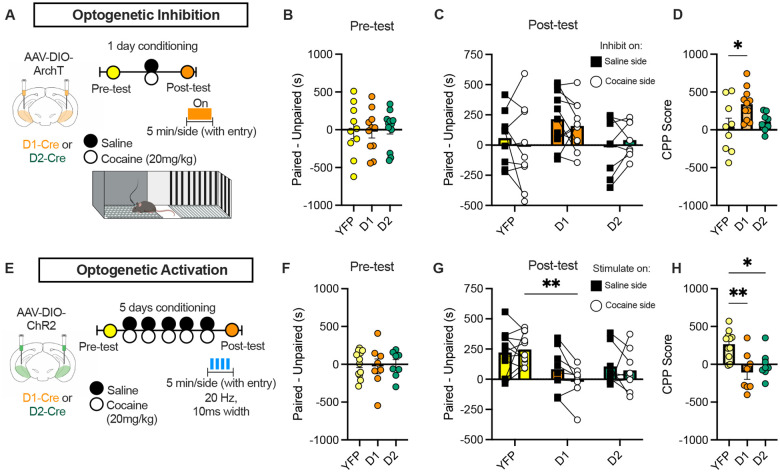
D1 activity is necessary for cocaine CPP. A) Schematic of optogenetic inhibition CPP experiment. B) Preference during pre-test before conditioning (YFP, *n=*9; D1-ArchT, *n=*11; D2-ArchT, *n=*8). C) Change in preference when inhibition is paired with entry to the saline or cocaine-paired side. D) Resulting average CPP score with inhibition (P=0.0255, One-Way ANOVA). E) Schematic of optogenetic activation (YFP, *n=*11; D1-ChR2, *n=*8; D2-ChR2, *n=*8). F) Preference during pre-test. G) Change in preference when stimulation is paired with entry to the saline or cocaine-paired side (P_Virus_ = 0.0120, 2-Way ANOVA). H) Average CPP score with activation (P=0.0015, One-Way ANOVA). Data represented as mean ± s.e.m

D1 and D2 cells in the striatum have been classically associated with opposing roles in reward processing, with D1 and D2 cells being linked to pro-reward and anti-reward roles, respectively^8,12^. A similar functional dichotomy has recently been suggested in the vHPC with respect to anxiety-like behavior, where manipulation of D1 and D2 neurons yields opposite effects on exploration under uncertainty^16^. To test whether vHPC dopaminoceptive populations similarly exert opposing control over reward memory, we used an optogenetic approach to selectively manipulate these cells during the expression phase of CPP (post-test) (Fig. 3). AAV vectors expressing the Cre-dependent inhibitory opsin, archaerhodopsin (ArchT), excitatory opsin (channelrhodopsin; ChR2), or YFP as a control were bilaterally injected into the vHPC of D1-Cre or D2-Cre mice, with fiber optics implanted directly above the injection site to allow for manipulation of D1 or D2 cells, respectively. Light stimulation was timed to the entry of the animal into the cocaine- or saline-paired compartment, allowing for unbiased, within-subject controls.

Given that we observe decreased D1 activity following CPP expression, we hypothesized that D1 cell inhibition after learning would enhance conditioned preference. Using a subthreshold cocaine CPP protocol, we observed that optogenetic inhibition of D1, but not D2, vHPC neurons was sufficient to drive robust CPP expression (Fig. 3A–C). In parallel, we used ChR2 to optogenetically activate D1 or D2 cells following our 5-day conditioning paradigm that we have shown induces robust CPP in D1-Cre and D2-Cre mice (Fig. 2B–C; 3E). Optogenetic activation of D1 cells blocked the expression of CPP (Fig. 3F–H). Together, these results demonstrate that inhibition of vHPC D1 cells is both necessary and sufficient for cocaine CPP expression.

Surprisingly, D2 activation also suppressed expression of CPP, despite no changes in our photometry signal across cocaine-context learning (Fig. 2I–M). Based on previous findings that D2 cell activity drives exploratory behavior^16^, we hypothesized that D2 stimulation may produce a context-independent, pro-approach signal that interferes with conditioned behavior. To directly test this possibility, we assessed the effects of D2 stimulation on real time place preference (RTPP) (Fig. S5). Optogenetic activation of vHPC D2 cells induced RTPP, whereas D1 stimulation had no effect, suggesting that D2 activation is intrinsically rewarding or motivating (Fig. S5A-C). These findings reveal that vHPC D2 cells can drive reinforcement, while D1 cells lack intrinsic rewarding properties. Instead, D1 cell inhibition facilitates the expression of cocaine-context associations.

### Transcriptomic regulation in D1 versus D2 vHPC cells after cocaine CPP

**Figure 4.**
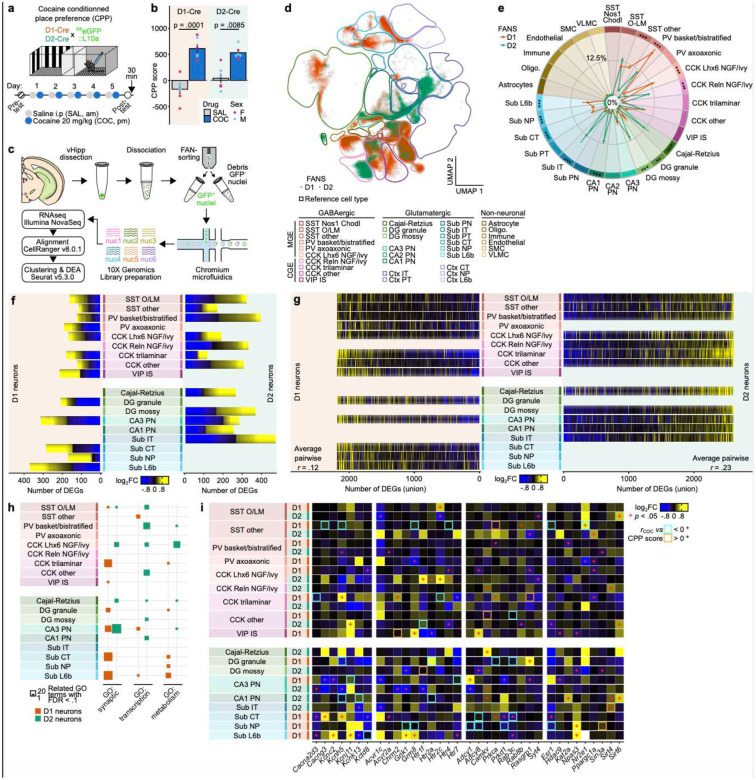
Cocaine CPP induces cell-type specific changes in transcriptome. A) Experimental design schematic. B) Preference in saline (control, *n=*6) and cocaine (*n=*6)-conditioned mice. C) Schematic of experimental design for single-nuclei RNA-sequencing after CPP. D) UMAP embedding of D1 and D2 cells across cell-types. E) Distribution of cell-types from sorted vHPC D1 and D2 cells. F) Differentially expressed genes (DEGs) across subpopulations of D1 and D2 cells. G) Heatmap of DEGs. H) Number of gene ontology terms associated with DEGs from each subpopulation that fit into synaptic, transcription, or metabolism category. I) Cell-type–specific selected genes across D1 and D2 classes; p-value indicates DEG and significant correlations with CPP score indicated.

Learning-associated, circuit-level plasticity mechanisms are largely supported by activity-dependent yet longer-lasting transcriptional programs^22,23^. In order to dissect the molecular mechanisms mediating CPP expression in D1 and D2 vHPC cells with the necessary cell type resolution, we used single-nuclei RNA-sequencing (snRNA-seq) (Fig. 4A–C) after CPP training. vHPC D1 and D2 nuclei were fluorescent activated nuclei (FAN)-sorted, sequenced and analyzed according to established methods^16^. First, we computationally and unbiasedly mapped our D1+ and D2+ nuclei to canonical hippocampal cell types by projection onto a published reference single-cell transcriptomic dataset from the Allen Brain Institute^24^ (Fig S6; see Methods for details). This largely recapitulated (Fig. S6–8) our initial transcriptomic study of these D1 and D2 vHPC cells^15^: virtually all nuclei were neuronal, and we similarly found large segregation of D1 and D2 cells across hippocampal cell types: i.e., more D1 than D2 parvalbumin (PV) interneurons, exclusively D2 hilar mossy cells, and dense D2 in CA1/subiculum glutamatergic neurons. However, this new reference-based analysis pipeline greatly refined cell type classification and therefore allowed for the detection of rarer (in terms of D1/D2 cells) cell types (i.e., vasoactive intestinal peptide (VIP) interneurons, D1 DG granule cells – consistent with histology) and for the finer segregation of pyramidal cell types, especially subicular cell types, into canonical, projection-defined, classes^24^. We found that D2 glutamatergic cells were comprised almost exclusively of CA1 hippocampal pyramidal neurons and subicular intratelencephalic (IT)-like neurons, while D1 glutamatergic cells were split across cortiocothalamic- (CT-), near projecting- (NP-), and layer 6b-like (L6b) transcriptomic classes. These findings: 1) show that the D1/D2 dichotomy cleanly map onto otherwise defined glutamatergic cell classes, confirming its relevance in distinguishing hippocampal glutamatergic neurons; and 2) suggest that D1 and D2 cells are segregated by projection patterns, with D2 cells (CA1 pyramidal, IT-like) projecting at long distance (to other neocortical areas or striatum), while D1 cells might project more locally (NP-like, L6b-like) or target the thalamus (CT). Finally, we also confirmed large transcriptional differences between D1 and D2 cells that extend beyond *Drd1* or *Drd2* expression (Fig. S8A-C), consistent with our initial study^16^.

Next, we probed cell-type-specific transcriptional regulation after cocaine CPP, with saline CPP as a control. Cocaine CPP elicited robust differential gene expression in vHPC dopaminoceptive populations across hippocampal cell types (Fig. 4F, Fig. S7D-E). We further found that these programs were widely distinct between D1 and D2 cells, including between D1 and D2 cells of the same hippocampal cell subtypes (Fig. S8D-E), but on the contrary were somewhat similar (i.e., positively correlated, with conserved heatmap patterns) between D1 cells of different hippocampal subtypes on the one hand and between D2 cells of different hippocampal subtypes on the other (Fig 4G, Fig S8F). This observation indicates shared D1 ensemble versus shared D2 ensemble responses to cocaine CPP, and reinforces the idea that the D1/D2 identity of a hippocampal neuron in large part dictates its transcriptional response to cocaine-associated learning over its hippocampal subtype identity: in other words, GABAergic and glutamatergic (for instance) D1 cells display regulation of similar sets of genes after cocaine CPP, and very different ones than D2 cells.

Gene ontology analyses of these cell-type-specific cocaine-induced transcriptomic changes identified that D1 differentially expressed genes (DEGs) were more heavily involved in synaptic biology and plasticity, while D2 DEGs were more involved with baseline neuronal processes like transcription (Fig. 4H, with selected example DEGs in Fig. 4I). These D1-selective, plasticity-related transcriptional differences could represent molecular substrates of the *ex vivo* electrophysiological (Fig. 2O–M) and *in vivo* functional (Fig. 2) differences observed above. For instance, we find that D1 rather than D2 CA1/subicular neurons regulate diverse calcium and potassium channels subunits (e.g., *Cacna2d3*, *Kcnc2*, *Kcnh5*) and signal transduction proteins (e.g., *Prkca*, *Prkd1*, *Rab3c*), which together could help explain increased excitability and distinct activation patterns.

Together, these data support cell-type-specific transcriptional reprogramming following cocaine CPP. These genes could represent potential molecular pathways underlying drug-context associative learning mediated by vHPC dopaminoceptive neurons, which stand as intriguing candidate targets for further mechanistic study.

## Discussion

Here, we further characterize the precise role of dopaminoceptive cells in the vHPC in behavioral control in the context of learning and plasticity, extending our previous work on their role in baseline exploratory behaviors^16^. We reveal that D1 cell activity is inversely related to valence, regardless of modality, with rewarding contextual cues decreasing their activity, while aversive auditory cues enhance their activity. This latter effect is consistent with a pro-avoidance role hypothesized in our previous work^16^. D2 cells, on the other hand, respond to unconditioned stimuli and are not dynamically altered across learning. As such, D1 cell inhibition drives memory recall of reward-context associations. Cocaine-induced transcriptional changes encode these distinct functions, with D1 cells undergoing the greatest gene expression changes related to synaptic function. Together, results of the present study establish that vHPC D1 versus D2 dopaminoceptive cells support distinct roles to drive motivated behavior after contextual associative learning and reveal potential molecular mechanisms driving their function.

Growing evidence supports a role for the vHPC in mediating the expression of conditioned drug seeking. Pharmacological inhibition or lesion studies have shown that the vHPC is critical for drug-, context-, and cue-induced reinstatement of cocaine^25–27^, alcohol^28^, and morphine^29^ seeking CPP and other paradigms^25–27,30,31^. In parallel, electrical stimulation of vHPC at 20 Hz has been shown to enhance cue-induced drug seeking after extinction^32,33^ or voluntary abstinence^34^. In contrast, our data demonstrate that optogenetic activation of vHPC D1 or D2 cells at the same frequency suppresses CPP expression. Our data indicate that these shared effects arise from distinct mechanisms: D1 cell activation seems to disrupt memory recall necessary for expressing learned preference, while D2 activation promotes a generalized reinforcing signal that interferes with expression of cocaine-context associations. These findings underscore that DA-sensing cells in the vHPC contribute to behavior through distinct and dissociable mechanisms, consistent with circuit-level studies showing opposing behavioral effects depending on the specific vHPC cell type or projection engaged^35,36^. It also reinforces the idea that vHPC dopaminoceptive cells constitute a particular sub-network within the vHPC.

However, the extent to which D1 and D2 populations contribute to the acquisition of drug-context associations remains to be determined. We do show that D1 cells are activated pharmacologically by a single cocaine exposure (Fig. 1). It has been shown that D1 receptor signaling in the vHPC is necessary for morphine CPP as well^29^, yet whether this role reflects memory formation, consolidation, or recall is unclear. Previous evidence has also demonstrated that D2 receptors in the vHPC are necessary for drug-induced reinstatement of morphine CPP^29^, and D2 receptors in the hippocampus have been demonstrated to modulate long-term plasticity, and spatial and recognition learning and memory^37^. Future studies will need to dissect the exact role of vHPC D1 and D2 cells in the acquisition of cue- or context-outcome encoding, as well as how these populations influence voluntary drug intake.

A central question that emerges from these findings is how vHPC D1 and D2 neurons drive distinct aspects of motivated behavior. One possibility is that they differ in their downstream connectivity. This is hinted at based on their transcriptomic make-up (Fig. 4). Prior studies have shown that projections from the vHPC to the nucleus accumbens (NAc) are sufficient to support reinforcement^38^. It remains to be determined whether vHPC D1 and D2 cells differentially engage such output pathways to mediate their respective behavioral effects. Another factor to consider is stimulation patterns: while 20 Hz electrical stimulation of the vHPC enhances cue-induced reinstatement of drug-seeking^32–34^, lower frequency (2 Hz) stimulation has no such effect, instead facilitating drug-primed reinstatement^39^. Similarly, recent work has shown that optogenetic stimulation parameters can drive opposing outcomes in reinforcement learning, CPP expression, and downstream activity in striatal D1 and D2 populations^8^. These findings raise the possibility that the behavioral effects of vHPC dopaminoceptive cells may depend not only on cell identity (D1 versus D2) and projection targets, but also on the temporal dynamics of their activation. Finally, our snRNA-seq data confirm that these vHPC DA-sensing cells represent heterogeneous neuronal populations, such that differences in excitatory versus inhibitory cell recruitment – or subtypes thereof – may also contribute to their opposing roles in goal-directed behavior and contextual memory expression through local microcircuit effects.

The classical model of striatal DA signaling posits that D1- and D2-expressing medium spiny neurons mediate opposite effects on reward, with D1 neurons promoting reinforcement and action initiation, and D2 neurons associated with aversion and behavioral suppression^8,12^. However, recent evidence from the NAc has challenged this simple dichotomy, suggesting instead that D1 and D2 MSNs act in concert to encode motivational salience, behavioral flexibility, and cue-outcome associations^6–9,11,12,40^.

Our findings suggest that this functional relationship does not extend uniformly across brain regions. In the vHPC, D1 and D2 neurons appear to operate as dissociable yet cooperating populations: they are activated by distinct environmental contingencies and contribute differently to motivational, emotional, and mnemonic processes. These results reveal a region-specific organization of dopaminoceptive signaling, in which vHPC D1 and D2 cells support separable components of associative learning and reinforcement, in ways that also differ from other vHPC circuits. Together, these data support a broader framework in which dopaminoceptive cells across the mesocorticolimbic system exhibit molecular, anatomical, and functional heterogeneity, govern behavior across timescales, and are particularly vulnerable to dysregulation in psychiatric conditions.

## Methods

### Subjects

All studies were carried out in accordance with the National Institutes of Health guidelines for Association for Assessment and Accreditation of Laboratory Animal Care accredited facilities. All experimental protocols were approved by the Institutional Animal Care and Use Committee at Mount Sinai. Male and female mice were maintained on a 12:12 hour light/dark cycle (07:00 lights on; 19:00 lights off) and were provided with food and water *ad libitum*. Wild-type (C57BL/6J; Jackson Laboratory) were used for viral-mediated knockdown of D1 or D2 receptors. Transgenic mouse lines (D1-Cre: MGI:3836633, D2-Cre: MGI:3836635, ^fl/fl^eGFP::L10a: IMSR_JAX:022367) were bred in-house on a C57BL/6J background. For fiber photometry experiments, D1-Cre or D2-Cre mice not crossed to eGFP::L10a were used; these mice were bred in house or at Charles River.

### Surgeries

Mice were anesthetized with an intraperitoneal injection of ketamine (100 mg/kg) and xylazine (10 mg/kg), then head-fixed in a stereotaxic apparatus (Kopf Instruments). Syringe needles (33G, Hamilton) were used to infuse 1 μl of virus at a 0.1 μl/min flow rate unilaterally for fiber photometry experiments, or bilaterally for all other experiments. Needles were kept in place for 10 minutes after injection before being retracted to allow for virus diffusion. Coordinates for vHPC were as follows, from bregma: AP −3.5 mm, ML +2.9 mm, DV −4.6 mm, 0° angle. For fiber photometry, 400 μm-wide optical fibers (Doric, MFC_400/430– 650 0.66_4.5 mm_MF2.5_FLT) were unilaterally implanted at AP −3.5 mm, ML +2.9 mm, DV −4.4 mm, 0° angle. For optogenetics, 200 μm-wide optical fibers (Doric, MFC_200/240–0.22_4.5 mm_MF1.25_FLT) were bilaterally implanted above vHPC at AP −3.5 mm, ML +2.9 mm, DV – 4.3 mm, 0° angle. Optical fibers were secured in place using dental cement (3M) and covered with a layer of black dental cement (C&B Metabond). Virus infusion and optical fiber placement was confirmed either by immunohistochemistry on fixed brains sections or by dissection of fresh tissue under fluorescent light. Mice were given at least 5 weeks recovery following surgery to allow for viral expression.

### Drug treatments

Cocaine was obtained through NIDA and diluted in saline and injected intraperitoneally at 20 mg/kg. Saline injections were used as a control.

### Viral reagents

For fiber photometry experiments, D1-Cre or D2-Cre mice were unilaterally injected with AAV9-CAG-FLEX-jGCaMP8f (Addgene, #162382-AAV9) in the vHPC. For optogenetics, D1-Cre or D2-Cre mice were bilaterally injected with AAV9-EF1a-DIO-hChR2(H134R)EYFP-WPRE-HGHpA (Addgene, #20298), AAV9- EF1a-DIO-EYFP (Addgene, #27056). No effect of genotype (D1-Cre versus D2-Cre) was observed in YFP conditions and data was collapsed into one control group. For viral-mediated knock-down of D1 or D2, wild-type mice were bilaterally injected with the AAV9-GFP-U6-m-DRD1A-shRNA (Vector Biolabs, #shAAV-257449) and AAV9-GFP-U6-m-DRD2-shRNA (Vector Biolabs, #shAAV-257451).

### FOS immunohistochemistry

D1- or D2-Cre^fl/fl^ eGFP::L10a mice were habituated to saline injections for 3 days. On the fourth day, mice were injected with cocaine (20 mg/kg; i.p) or saline, and placed in locomotor boxes for 1 hour. Immediately after this one hour, mice were transcardially perfused with PBS and then 4% paraformaldehyde (PFA). Brains were post-fixed for 24 h in 4% PFA at 4°C. Sections of 40 μm thickness were cut in the coronal plane with a vibratome (Leica) and stored at 4°C in PBS. Slices were stained for GFP to boost native signal (anti-GFP primary antibody; chicken polyclonal; 1:500; #GFP-1020, Aves), and FOS (anti-FOS primary antibody; rabbit polyclonal; 1:500, Abcam) overnight. Slices were incubated with anti-chicken Alexa Fluor Fluor^®^-488-conjugated (goat; 1:500; #AB150169, Abcam), anti-rabbit Alexa Fluor^®^-594-conjugated (goat; 1:500; #AB150080, Abcam), counterstained with DAPI, and mounted on microscope slides in ProLong Gold Antifade Mountant (ThermoFisher Scientific). Confocal images were acquired on a LSM880 laser scanning confocal microscope (Zen Black v2.3). 5–7 whole hippocampal images were taken per animal and 6×7 images were stitched.

### Image processing and cell segmentation

Multi-channel fluorescence images were processed using a custom R pipeline (R v 4.5.1) largely relying on the following packages: EBImage v4.50.0, sf v1.0–21, terra v1.8–60, reticulate v1.43.0, imager v1.0.5, FNN v1.1.4.1, concaveman v1.1.0, tidyverse v2.0.0. All analyses were performed with parallel processing optimization and memory management using future v1.67.0 when possible for batch processing of multiple images, and are briefly described below.

#### Image preprocessing.

Raw multichannel TIFF images were first standardized via pixel size calibration (to 0.3785 μm/pixel). Each channel underwent sequential preprocessing steps: (1) top-hat filtering using a disc-shaped structuring element (radius = 10 pixels) to remove background fluorescence and enhance cellular signals, (2) median filtering (kernel size = 3 pixels) to reduce noise while preserving edge information. Preprocessed single-channel images were saved as 8-bit TIFF files for downstream analysis.

#### Cell segmentation.

Automated cell segmentation was performed using the *Cellpose v4.0.6* deep learning framework (PMID: 33318659, https://doi.org/10.1101/2025.04.28.651001) via the *reticulate* package interface to *Python v3.10.18* and relying on *numpy v2.2.6*. The pre-trained CellposeModel “cpsam” was applied to channels 1 (FOS) and 2 (GFP) with GPU acceleration enabled. Segmentation parameters included: flow threshold = 0.4, cell probability threshold = 0, minimum cell size = 30 pixels, with diameter estimation enabled and without percentile-based intensity normalization. Segmented regions of interest (ROIs) were exported for visualisation as ImageJ-compatible ZIP files using the *roifile v2025.5.10* Python package.

#### ROI analysis and filtering.

For each detected ROI, morphological and intensity metrics were calculated. ROI polygons were processed using the *sf* package for spatial operations. Shape descriptors included area, centroid coordinates, circularity (4π × area / perimeter^2^), aspect ratio, and solidity (area/convex hull area). Intensity measurements comprised mean and median values for both the ROI interior and a 3-pixel contour background region, calculated using *terra* package raster operations.

Quality control filtering and valid cell ROI selection were applied based on morphological criteria: circularity > 0.4, aspect ratio < 3, solidity > 0.6, and area between 80–1000 pixels. Additional channel-specific intensity thresholds were applied (channel 1 for FOS-positive cells: median ROI intensity > 10, signal above background > 0.03 × 255 (i.e. 3% of the full 8-bit grayscale); channel 2 for GFP positive cells: median ROI intensity > 10).

#### Co-localization analysis.

Spatial overlap between channels was assessed using computational geometry methods implemented in the *sf* package. ROI polygons were converted to spatial feature objects, and pairwise intersections were first computed for bounding box pre-filtering to reduce computational load, followed by precise geometric overlap calculations via *st_intersection()*. Overlap metrics included intersection-over-union (IoU), fractional overlap relative to each ROI area, and absolute overlap area. Double-positive cells were defined as ROI pairs with fractional overlap > 80% in channel 1, i.e. cells for which >80% of the FOS ROI was comprised within a GFP ROI.

#### Regional segmentation and mapping.

Brain region boundaries were defined using manually registered segmentation masks processed with *EBImage*. Masks were binarized and labeled using *imager*. Region contours were refined using a custom concave hull algorithm (*concaveman* package) to define outer boundaries, followed by label propagation via k-nearest neighbor assignment (*FNN* package) to fill gaps up to 100 pixels from labeled regions. Small regions (<2000 μm^2^) were filtered as artifacts. All segmentation masks were inspected and labelled with atlas region names manually. Cell-to-region assignment was performed by mapping ROI centroids to the labeled region mask using pixel-wise lookup. Final cell classifications included single-positive (channel 1 (FOS) or 2 (GFP)) and double-positive categories, with regional localization data.

### Cocaine conditioned place preference

CPP was performed in a three-chamber apparatus consisting of two side chambers measuring 28 × 24 cm each, and a center chamber measuring 11.5 × 24 cm. One side had black and white stripes on the walls with a metal grid floor, and the other had gray walls with a punched metal floor. An unbiased CPP paradigm was used in the current study.

On day 1, mice were allowed to explore the entire apparatus for 20 minutes (pre-test). On conditioning days (day 2–6 for most experiments except optogenetic inhibition with ArchT where only one day of conditioning was performed), mice were confined to one of the chambers and injected with saline in the morning, cocaine (20 mg/kg) in the afternoon. At least four hours were given between the morning and afternoon conditioning sessions. On the last day (day 7, except for optogenetic inhibition experiments where it is day 3), mice were allowed to freely explore apparatus in a choice test (post-test). Preference for the chamber was compared with day 1; CPP score is calculated as: time spent in the cocaine-paired side on the post-test – time spent in the cocaine-paired side on the pre-test. Paired – Unpaired Time refers to time spent on the cocaine-paired compartment – time spent on the saline-paired compartment.

For optogenetic stimulation/inhibition experiments, LED stimulation was time-locked to entry to either compartment (saline or cocaine-paired) for 5 minutes at a time, counterbalanced between sides. If mice did not enter that side during the five minutes, there was no LED stimulation. For fiber photometry experiments, recordings of somatic D1 or D2 cell activity were conducted during pre-test and post-test. For snRNA-seq and electrophysiology, control mice refer to mice conditioned only with saline in both the morning and afternoon sessions.

### Nuclei purification, fluorescence-activated nuclei sorting (FANS), and single-nuclei RNA-sequencing following cocaine CPP

D1- or D2-Cre^fl/fl^eGFP::L10a mice underwent cocaine (or saline as control) conditioned place preference paradigm as described above with 5 days of conditioning. 30 minutes after the start of the posttest, mouse brains were collected following cervical dislocation and followed by rapid bilateral vHPC dissections from 1 mm-thick coronal brain sections and stored at −80°. Samples were prepared as previously described^16^.

#### Data processing and analysis.

All analyses were performed in R v4.5.1 using the following key packages: *Seurat v5.3.0* for single-cell analysis, *DESeq2 v1.48.2* for bulk RNA-seq analysis, *DoubletFinder v2.0* for doublet detection, *lmerTest v3.1–3* for mixed-effects modeling, *clusterProfiler v4.16.0* for functional enrichment, and custom visualization functions built on *tidyverse v2.0.0* and *stats v4.5.1* related packages. When possible, parallel processing was implemented using the *future* package with multicore plans (typically 4–8 workers) to accelerate computationally intensive steps while maintaining reproducibility through *set.seed()* operations.

#### Reference dataset processing.

Single-cell RNA sequencing data from mouse hippocampus and associated regions were obtained from the Allen Brain Atlas^24^, specifically using 10X Genomics processed nuclei from hippocampal formation (HIP), parahippocampal regions (PAR-POST-PRE-SUB-ProS), and entorhinal cortex (ENT). Gene expression matrices and associated metadata were imported from HDF5 format using the *rhdf5* package and converted to *Seurat v5* objects. Cell type annotations were filtered to retain only cells from hippocampal regions of interest, excluding cortical areas other than enthorinal cortex.

The reference dataset underwent *SCTransform* normalization (vst.flavor=“v2”). Principal component analysis (PCA) was performed on the top 50 dimensions, followed by UMAP dimensionality reduction with min.dist=1.5 and spread=1.5 parameters, with return.model=TRUE to enable projection of query datasets. Original cell type annotations and UMAP coordinates from the source publication were preserved, yet the original 343 cell types in the reference dataset were manually combined into 33 new more general and biologically meaningful cell type labels for clarity and interpretability throughout analysis and visualization.

#### Experimental dataset processing.

Raw 10X Genomics count matrices from experimental samples were imported using the *Read10X* function and converted to individual Seurat objects. Quality control filtering was applied with the following thresholds: 1,000–30,000 total UMI counts per cell, 500–6,000 detected genes per cell, and <1% mitochondrial gene expression. Each sample underwent *SCTransform* normalization (vst.flavor=“v2”) with mitochondrial gene percentage included as a regression variable (vars.to.regress=“percent.mt”) and retaining all genes for downstream analysis. Principal component analysis was performed on the top 50 dimensions for each sample independently. Putative doublets were identified using *DoubletFinder v2.0* with the following parameters: expected doublet rate of 7.5% based on cell recovery numbers, PCs 1–50 for neighborhood construction, and pN=0.25 for artificial doublet generation. Optimal pK values were determined through parameter sweeping using *paramSweep* and *find.pK* functions, with the *BCmetric* used to identify the peak. Doublets were tagged with classifications but retained for initial analysis, allowing for downstream filtering decisions.

#### Cell type annotation via reference mapping.

Query datasets were mapped to the reference atlas using Seurat’s anchor-based integration workflow with the following specifications. Transfer anchors were identified using *FindTransferAnchors* with normalization.method=“SCT”, reduction=“pcaproject”, reference.reduction=“pca”, and dims=1:50. Only genes present in both reference and query datasets were used as features for anchor finding. Cell type annotations were subsequently transferred using *MapQuery*, which projects query cells into the reference UMAP space using the pre-computed reference PCA and UMAP models. This approach assigns cluster labels based on weighted nearest neighbor analysis in the shared embedding space. The reference cell type hierarchy was then mapped onto query cells, with prediction scores calculated to assess mapping confidence. Additional metadata including hierarchical cell type classifications (both original and new cell type labels) were propagated from the reference annotations to provide multiple levels of cellular identity resolution.

#### Quality control and post-mapping filtering.

A multi-stage filtering approach was implemented for final dataset curation. First, only cells identified as “Singlet” by DoubletFinder classifications were retained, removing all cells flagged as doublets. Second, putative cortical contamination was removed by excluding cells assigned to enthorinal cortical cell types. Final quality control metrics were calculated at each filtering stage, including cell counts per sample, median UMI and gene counts, median mitochondrial gene percentages, and median prediction scores for cell type assignments. Sample-level metrics were exported to ensure balanced representation across experimental conditions.

#### Differential expression analysis.

For comprehensive gene expression analysis, individual sample Seurat objects were merged using a two-step approach to manage memory constraints. First, all assays except SCT were merged using standard Seurat merge functions with add.cell.ids to maintain sample identity. Subsequently, SCT assays were merged separately and integrated into the combined object. Cell identifiers were prefixed with sample names to ensure unique cell barcodes across the merged dataset.

#### Cell-level analysis using MAST.

Cell type marker genes were identified using Model-based Analysis of Single-cell Transcriptomics (MAST) implemented through Seurat’s *FindAllMarkers* function. The analysis used the SCT assay with the following parameters: min.cells.group=3, logfc.threshold=log2(1.5), min.pct=0.3, and only.pos=TRUE to identify positive markers. Latent variables including donor sex, drug treatment, and sample identity were included to control for technical and biological confounders.

For D1 versus D2 dopamine receptor subtype comparisons, differential expression was performed using FindMarkers with MAST across all cells combined with cell type identity included as a latent variable. The analysis used the SCT assay with min.pct=0 and logfc.threshold=-Inf to capture the full dynamic range of expression differences, while controlling for donor sex, drug treatment, and sample identity as latent variables.

#### Sample-level pseudobulk analysis.

For cocaine versus saline drug treatment comparisons, a pseudobulk approach was implemented to account for sample-level variation and avoid pseudoreplication. Raw UMI counts from the RNA assay were aggregated using *AggregateExpression* by sample, cell type, D1/D2 subtype, sex, and drug treatment combinations. Only samples with ≥25 cells per cell type and cell types with ≥3 samples per drug condition were included in the analysis.

Pseudobulk count matrices were analyzed using DESeq2 with the design formula ~drug + sex, where drug treatment served as the primary factor and sex as a covariate. DESeq2 normalization and variance stabilization were applied using default parameters with independentFiltering=FALSE to retain all genes for downstream analysis. Differentially expressed genes were identified using the following criteria: baseMean ≥75th percentile within each dataset, fold change > 15%, and nominal P-value < 0.05. The 75th percentile baseMean threshold was calculated separately for each cell type to account for cell type-specific expression levels.

#### Functional analysis and pathway enrichment.

Gene Ontology (GO) enrichment analysis was performed using the *clusterProfiler* package (v4.x) with the mouse genome database (org.Mm.eg.db). Gene symbols were converted to Entrez IDs using the *bitr* function, and enrichment analysis was conducted using enrichGO with the following parameters: ont=“BP” (biological processes), pAdjustMethod=“fdr”, pvalueCutoff=0.1, qvalueCutoff=0.1, and minGSSize=20. The background universe was defined as all expressed and detected genes (baseMean > 0) within each respective cell type to avoid bias toward highly expressed gene sets. Additional filtering required ≥3 genes per significant term, with false discovery rate correction applied within each analysis.

Upstream regulator analysis was conducted using Ingenuity Pathway Analysis (IPA, QIAGEN) on differentially expressed gene lists. Input data included gene identifiers, baseMean expression values, log2FoldChange values, P-values, and binary differential expression status. Activation z-scores were calculated based on the directional consistency between observed gene expression changes and known regulatory relationships in the IPA knowledge base. Significant upstream regulators were defined using IPA’s default statistical thresholds with additional filtering for ≥3 target molecules per regulator.

#### Visualization and custom analysis functions.

Differential expression results were visualized using custom heatmap functions with hierarchical clustering (method=“ward.D2”) and principal component analysis for gene ordering. Correlation analyses between cell types used Pearson correlation coefficients. Radar plots for cell type distributions were generated using *ggradar* with custom functions to display mean ± standard error for each condition.

#### Behavioral correlation analysis.

Normalized pseudobulk expression values from DESeq2 analysis were correlated with conditioned place preference (CPP) behavioral measures using Spearman correlation analysis (*cor.test* function). Behavioral metrics included pre-test scores, post-test scores, and difference scores (post-test minus pre-test) as measures of cocaine-induced place preference. Correlations were computed separately for cocaine-treated samples within each cell type and D1/D2 condition, with minimum sample sizes of n≥3 per group. Results were filtered to retain correlations with P < 0.05, and genes showing significant correlations in multiple cell types were prioritized for biological interpretation.

### Statistical analysis

Cell type distribution analysis was performed using linear mixed-effects models implemented in *lmerTest* with the formula: percentage ~ sorted_label [cell identity determined by Yao, 2021^24^] × donor_sex_label × drug_label × cell_type + (1|sample_ID). Post-hoc pairwise comparisons were conducted using *emmeans* with false discovery rate (FDR) adjustment for multiple comparisons. Degrees of freedom were calculated using the Kenward-Roger method to account for unbalanced designs.

### Fiber photometry recordings and analysis

For somatic recordings of D1 or D2 vHPC cell activity, the fiber photometry system (FP3002 system from Neurophotometrics, NPM) was time-locked with the video-tracking system (Ethovision XT 11, Noldus or ANY-Maze) via transistor-transistor logic signals (TTLs). Low-autofluorescent patchcords (Doric, MFP_400/430/1100–0.66_3m_FCM-MF2.5_LAF) were used according to manufacturer’s instructions with the FP3002 Bonsai node. Fluorescence signals resulting from 470 nm, 560 nm and 415 nm excitation, interleaved in time, were sampled at 78 Hz total, *i.e*. at a 26 Hz effective sampling rate for each channel. Analysis was performed as previously described^16^.

For analysis, deinterleaved NeuroPhotometrics time series data was directly exported from Bonsai. To compare neuronal activity across animals and behavioral sessions, individual animal time-series data were analyzed using custom R codes following published standard methods with minor modifications. Briefly, 470, 560 and isosbestic 415 nm (for NPM) signals were first smoothed using a 4th-order 5 Hz lowpass Butterworth filter built using the *gsignal v0.3–5::butter* function. To remove the bleaching slope and low- frequency fluctuations, baseline correction was then performed by subtracting the baseline obtained by regressing each individual signal using the LOWESS smoother (*stats::lowess*) with default parameters from the smoothed 470/560 and 415 nm signals. Both 470/560 and 415 nm signals were then standardized using a robust z-score (z(F)=(F–median(F))/mad(F)). The standardized 415 nm signal was then fitted to the standardized 470 nm signal using the robust regression function *MASS v7.3::rlm*, and normalized dF/F z(dF/F) was finally calculated as the difference between the 470nm signal and the fitted 415 nm signal to remove motion artifacts and autofluorescence. To analyze time-locked neuronal activity in respect to behavior, the normalized z(dF/F) signal was extracted around the onset of the relevant behavior (defined as t = 0 s). For compartment-based spatial analysis, signal changes were quantified for relevant time intervals (in each compartment) as the corresponding areas under the curve (the curve being the entire z(dF/F) time-series), which were calculated with linear interpolation using the *MESS v0.5.9::auc* function. For peri-event analysis, signal trace data are quantified and averaged with n = event, and signal changes are measured as the area under the curve (AUC) within relevant time intervals. For entries, only full transitions (saline -> center -> cocaine, or cocaine -> center -> saline) were used.

### Ex vivo slice electrophysiology

D1-tdtomato+ mice were deeply anesthetized with isoflurane and decapitated. Brains were rapidly removed and chilled in artificial cerebrospinal fluid (ACSF) containing (in mM): N-methyl-D-glucamine 93, HCl 93, KCl 2.5, NaH2PO4 1.2, NaHCO3 30, HEPES 20, glucose 25, sodium ascorbate 5, thiourea 2, sodium pyruvate 3, MgSO4 10, and CaCl2 0.5, pH 7.4. The brain was embedded in 2% agarose and coronal slices (200 μm thick) were made using a Compresstome (Precisionary Instruments). Brain slices were allowed to recover at 33 ± 1°C in ACSF solution for 30 min and thereafter at room temperature in holding ACSF, containing (in mM): NaCl 92, KCl 2.5, NaH2PO4 1.2, NaHCO3 30, HEPES 20, glucose 25, sodium ascorbate 5, thiourea 2, sodium pyruvate 3, MgSO4, and CaCl2 2, pH 7.4. After at least 1 h of recovery, the slices were transferred to a submersion recording chamber and continuously perfused (2–4 mL/min) with ACSF containing (in mM): NaCl 124, KCl 2.5, NaH2PO4 1.2, NaHCO3 24, HEPES 5, glucose 12.5, MgSO4 2, and CaCl2 2, pH 7.4. All the solutions were continuously bubbled with 95% O2 / 5% CO2. vCA1 or vSub pyramidal cells were visually identified with infrared differential contrast optics (BX51; Olympus) and fluorescence visualized through eGFP bandpass filters upon LED illumination through the objective (p3000^ULTRA^, CoolLed) using μManager v2.0. Whole-cell patch-clamp recordings were performed at room temperature using a Multiclamp 700A amplifier (Molecular Devices). Recording electrodes (3–5 MΩ) pulled from borosilicate glass were filled with solution containing (in mM): K-gluconate 122, HEPES 10, KCl 5, MgATP 5, Na2GTP 0.5, QX314 1, and EGTA 1, pH 7.25. Data acquisition (filtered at 10 kHz and digitized at 10 kHz) and analysis were performed with pClamp 11 software (Molecular Devices). Following the breakthrough cells were allowed to stabilize for 3 min before the recordings were made. Neurons were current clamped at I = 0 and baseline membrane potentials were recorded.

### Optogenetics

For optogenetic stimulation experiments, mice were connected to a dual optical fiber patchcord (Doric) connected to a 473 nm blue laser (OEM Laser System). Stimulation was executed in the form of 10 ms box 900 pulses emitted at 20 Hz with an output power of ~8 mW at the tip of the fiber. For optogenetic inhibition experiments, mice were connected to a 565 nm laser, with an output power of ~8 mW.

### Statistics

Fiber photometry statistics were performed in R v4.2.2 mostly relying on 920 *stats v4.0.2*, *tidyverse v1.3.1* and *lmerTest v3.1–3* packages. In summary, pairwise comparisons were performed with Welch’s *t*-tests (*stats::t.test* function), correlations using Pearson’s *r* (*stats::cor.test* function), independence testing with c^2^ tests (*stats::chisq.test* function) and more complex multifactorial designs were analyzed using linear models computed with the *stats::lm* function for fixed effects-only models or *lmerTest::lmer* function for mixed effects models. Random effects (conceptualizing non- 925 independent observations, such as repeated measures and/or nested observations) were modeled as random intercept factors. Subsequent analysis of variance (LM/LMM-ANOVA) was performed using type III sums of squares with Kenward-Roger’s approximation of degrees of freedom. Post-hoc testing was performed using the *emmeans* package and significance was adjusted for false discovery rate (FDR) at a 0.05 level using standard Benjamini-Hochberg procedure. The rest of the statistics were performed in Prism; pairwise comparisons performed with *t*-tests, or 2-Way ANOVA (compare 2 groups across 2 conditions). Bar and line graphs represent mean ± sem. Correlation graphs represent regression line with its 95% confidence interval. Significance was set at *p* < 0.05.

## Supplementary Material

This is a list of supplementary files associated with this preprint. Click to download.

- SupplementaryFigures.pdf

## Data Availability

All snRNAseq data reported in this study will be deposited in the Gene Expression Omnibus. All other data, including raw photometry data, are available upon request. *GEO dataset will be made public upon publication - for review, please email the corresponding author (*eric.nestler@mssm.edu*) for private access keys*.

## Code availability

Custom scripts and code utilized in this study, including for statistical analysis, are available upon request.
